# Secular Trends of Children’s Physical Fitness and the Impact of the COVID-Pandemic for Years 2012 to 2023

**DOI:** 10.1186/s40798-025-00881-2

**Published:** 2025-06-24

**Authors:** Tanja Eberhardt, Klaus Bös, Alexander Woll, Reinhold Kliegl, Claudia Niessner

**Affiliations:** 1 Institute of Sports and Sports Science, Karlsruhe Institute of Technology, Karlsruhe, Germany; 2 Division of Training and Movement Sciences, University of Potsdam, Potsdam, Germany

**Keywords:** Monitoring, Motor performance, Youth, COVID pandemic, Data pooling, Cohort study

## Abstract

**Background:**

Physical fitness is a key component in the development of an active lifestyle and a determinant of future health, particularly in childhood. The findings of physical fitness assessments enable evidence-based monitoring and the identification of long-term trends. The COVID-19 pandemic is an additional factor that can be considered in the analysis, as its influence is already known. The aim of our analysis was to comprehensively investigate secular trends with respect to the physical fitness of children before and during the pandemic. This study also serves to test whether Citizen Science projects can deliver results comparable to those obtained using traditional assessment formats while also documenting certain limitations of this approach.

**Methods:**

Data on annual assessments conducted in the German federal state of Baden-Wuerttemberg were pooled from 12 cohorts starting in 2012. The analyses are based on 25,580 6–10 year-old children (*M* = 7.56, *SD* = 1.21 years; 12,575 girls) in our analysis. We estimated the effects of the COVID-19 pandemic using a regression discontinuity design within a linear mixed model. This enabled us to estimate pre-pandemic and pandemic trends, and to adjust for age, sex, and body constitution fixed-effect covariates and child and region as random factors.

**Results:**

For the pre-pandemic cohorts, we found significant trends only slightly negative or positive trends in six of eight items. This suggests that the declining trend in physical fitness in children has been slowing down. The COVID-19 pandemic affected physical fitness negatively for six items (i.e., shift at critical date: 6 min Run, Jumping Sideways, Sit-Ups, Push-Ups; negative change from pre-pandemic to pandemic trends: 20 m Sprint, Standing Long Jump). There was no evidence for pandemic changes in balancing backwards and stand-and-reach items. Effects of age, sex, and body constitution replicated previous results.

**Conclusions:**

Continuous monitoring of children’s physical fitness is essential, especially in the context of the COVID-19 pandemic. Such monitoring identifies positive and negative trends and provides evidence for the need of strategies and actions. It is particularly important to initiate systematic initiatives during childhood to promote physical fitness and reduce deficits, as this is the time when the foundations for an active and healthy lifestyle are laid.

**Supplementary Information:**

The online version contains supplementary material available at 10.1186/s40798-025-00881-2.

## Background

Physical fitness (PF) consists of multiple dimensions that reflect a person’s ability to perform physical activities or physical exercise [1]. According to Caspersen et al. [2], PF can be categorized into health- and skill-related dimensions and “…being physically fit has been defined as “the ability to carry out daily tasks with vigor and alertness, without undue fatigue and with ample energy to enjoy leisure-time pursuits and to meet unforeseen emergencies”.”. It distinguishes between five main dimensions of PF: endurance, strength, speed, coordination, and flexibility (a passive dimension). These latent abilities can be measured through PF assessments comprised of different items. PF is one of the fundamental determinants for the development of a healthy and active lifestyle [1]. PF that has been acquired in childhood is the foundation on which more specific motor competencies and movement patterns develop [3]. It enables children to be physically active, also later in life, and thus helps to prevent negative health outcomes [4–6]. Findings also suggest that cardiorespiratory fitness, muscular fitness and muscular endurance remain moderately stable over a person’s lifetime [7, 8]. An improved level of cardiorespiratory fitness reduces the risk of cardiometabolic diseases later in life, as well as future disability [8, 9]. Higher muscular fitness is associated with a lower risk of obesity and adiposity deposition as well as skeletal health [10–12]. Moreover, the function of PF as a marker of health extends to cognitive and psychological benefits [13–15].

The assessment of PF in children is crucial given its health-related link, and more importantly because the foundations of PF are laid in childhood. Assessments enable evidence-based monitoring and provide insights into PF levels in youth populations. Long-term monitoring is even more essential so that PF changes and trends can be detected and results can be analysed and compared to previous and future findings [16]. There is already evidence for secular trends and the literature has found declines in PF in children for the past decades until the begin of the century. However, for the recent years these negative trends stagnated on a low level Tomkinson & Olds [17] recently summarised the findings for aerobic fitness between 1958 and 2003 and included data on over 25,000,000 children aged 6–19 years. Aerobic performance decreased −0.36% per annum over the entire measurement period. More specifically, the decline started after improvements began shifting in a negative direction in 1970. A more recent study on secular trends in cardiorespiratory endurance, based on a meta-analysis of data on the 20 m-shuttle run test, revealed that the decline in endurance has slowed and stabilised since 2000 [18]. This is in line with another review, which analysed national trends in all PF dimensions, and with findings from the national MoMo-study in Germany, which found a plateauing of endurance levels between 2003 and 2017 [19, 20]. A meta-analysis by Fühner et al. [21] found that, of the different dimensions of PF, cardiorespiratory endurance to have the largest effects of the different dimensions of PF in declining secular trends since 1986, but that it has remained stable since 2010. Changes in anaerobic performance, operationalised with speed, muscular power and strength tests, have been found to be smaller compared to secular changes in aerobic fitness [21, 22]. The global pattern of change was comparatively consistent and stable worldwide between 1958 and 2003 [22]. Recently, Tomkinson et al. [23] reported similar results with respect to performance in the standing long jump, which represents leg strength, as improvements had been negligible every decade since 1960. This finding has also been reported for relative muscle strength as the magnitude of the secular trends is minor and has been consistent [21]. The national MoMo study analysed population-based trends in PF by comparing three measurement time periods. Overall, the study revealed positive trends between the baseline (2003–2006) and wave 1 (2009–2012) but found a stagnation between wave 1 and wave 2 (2014–2017). The plateauing was identified for strength, speed, and coordination in the group aged 4–17 years [19, 24]. Overall, national and international research has found a “low level of stagnation for PF” [20, 25]

The spread of the COVID pandemic after 2020 affected the lives and circumstances of children. The limitations imposed by lockdowns and school closures disrupted daily routines and reduced opportunities for physical activity and social interaction. Recent studies indicate significant changes in the mental health of children, including issues such as anxiety and depression [26]. The COVID pandemic and its associated restrictions have significantly impacted activity behaviours in Germany as research has found a marked decrease in physical activity and an increase in media consumption among children [27]. Consequently, these changes also might have a substantial impact on children’s PF. Several studies examined this in the years following the COVID pandemic. Most of them compare two measurement time points or periods: before and after the pandemic. Declines in endurance, in particular, were identified after the COVID pandemic by several studies focusing on Europe and Asia [28–33]. Teich et al. [34] report changes for all six tested dimensions of PF and a decreased performance in the tests for endurance, speed and coordination. They classified pre-pandemic cohorts 2016–2019 and pandemic cohorts 2020–2022, and benefitted from the annual testing of third graders in Brandenburg which began in 2016. This enabled them to detect secular trends during the period and to consider COVID pandemic influences. Martinko et al. [33] also found that levels of PF dropped, however they differentiated between children’s weight and reported a smaller decrease in obese children. 

There are already some studies with a large size, a uniform sample, continuous measuring points, and systematic monitoring with a standardised assessment of PF for some countries, that use the same test profiles over time [33, 35–38]. However, monitoring requires a homogeneity and specificity of the different aspects in order to detect secular trends through surveillance and to take affected groups as well as specific situations and hotspots into consideration [16]. The evidence suggests identifying country-specific trends and more differentiated results for different age groups and dimensions of PF. 

Against this background using data from a convenience sample, we examined secular trends in children’s PF over a 11-year period since 2012, tested with a uniform measurement method and design. Our aim is to estimate trends in the different dimensions of PF and to identify changes specifically for the presented age-group and region. Because the COVID pandemic had a significant impact on children’s lives and, consequently, on their PF, the secular trend was modelled taking this particular influencing factor into account and assess potential changing effects on children’s PF.

## Methods

### Study Design

The data presented in this paper are drawn from the project “Fitnessbarometer” in cooperation with the Kinderturnstiftung of Baden-Wuerttemberg and therefore the sample is limited to this German federal state. The data on PF was collected between 2012 and 2023 based on the test profiles of the German Motor Test 6–18 (GMT) and Kinderturntest Plus 3–10 (KITT +) and using a dimension-oriented approach [39–41]. Tests were administered by practical experts, that is teachers, educators, coaches. In addition to their usually existing knowledge in physical education, they were trained and educated as multipliers with courses and material, which describes precisely the standardisation of assessment. Further, they received personal support for questions if necessary. After assessment, data was entered into an evaluation software. The authors received the raw data pseudonymised by year and uploaded it into MO|RE data, a sports-specific e-research infrastructure (www.motor-research-data.de, [42]). A set of plausibility and quality checks led to the selection of a subset of the data to be described below. For our analysis, data pooling was used to merge the individual cohorts into one large dataset comprising all cohorts from 2012 to 2023. Therefore, the paper used a cross-sectional cohort design with a convenience sample [43–51].

### Physical Fitness Tests

The test assesses latent dimensions of PF on a measurable skill level, operationalised with the following eight items: 6 min Run, 20 m Sprint, Standing Long Jump, Push-Up, Sit-Up, Jumping sideways, Balancing backwards and Stand-and-Reach. Each item represents at least one cell of a taxonomy of tasks (see Table 1). Four items (i.e., Standing Long Jump, Jumping sideways, Balancing backwards and Stand-and-Reach) were assessed for 3–5 year old children; all eight items were assessed for children aged 6–10 years. Constitutional data on height and weight were also collected. Detailed description of individual items is documented in the test manuals.

**Table 1:** Taxonomy with task structure and items of the GMT (modified by Bös et al., 2023)

Structure of Task	Motor Dimension					Passive Systems of Energy Transfer
		Endurance	Strength	Speed	Coordination	Flexibility
Locomotor Movements	Walking, Running	6 min Run		20 m Sprint	Balancing backwards	
	Jumping		Standing Long Jump		Jumping sideways	
Movements of Bodyparts	Upper Extremities		Push-Up			
	Trunk		Sit-Up			Stand-and Reach

### Data Processing

The different test protocol (i.e., four vs. eight items) necessitated separate analyses for the younger and older group of children. Here we report results only for 6 to 10 year-old children. The same procedures and analyses were carried out for the younger children and results for this group are documented in a Supplementary Material.

For data pre-processing and post-processing (i.e., construction of tables and figures) we used the R programming language (R Core Team, 2023) in the RStudio IDE, primarily packages *tidyverse* [52]*, easystats* [53] and *childsds* [54]. We used the *childsds package* to convert the raw scores of mass, height, and BMI [i.e., mass (kg)/height (m)^2^] into standardized age- and sex-adjusted z-scores (zMass, zHeight, zBMI) [54]. These z-scores (also known as BMI-SDS, Mass-SDS, and Height-SDS) were computed with reference to anthropometric tables of a large representative sample of German children using the LMS method [55–57]. Based on this, we excluded children with absolute SDS values ≥ 3 (yielding cubic terms < 7 and quadratic terms < 6) to ensure valid quality of data (lost 1,437 children).

In addition, to consider the specific circumstances of data collection and ensure validity, reliability, and objectivity of data, data processing was complemented by further checks of plausibility. Due to potential variation in instruction for some items at some events and cohorts, means were out of a meaningful range and showed an irregular number of high scores relative to representative norm values [58, 59] [66]. The deviations were apparent for sex x age cells at the event-cohort level. We identified cells with means outside the 5th and 95th percentile and excluded the scores of all children for the respective item at this event-cohort from the analyses (lost 10,184 children and 116,458 observations).

The 20 m Sprint score was converted to a speed score (i.e., 20/time), justified by a Box-Cox distribution analysis. With this transformation higher values also imply higher fitness for all scores. All scores were converted to z-scores to facilitate comparisons between item, sex, and age effects. Z-scores outside of a ± 3 SD range were excluded within sex x age cells and recomputed without outliers (lost 5 children and 610 observations).

This left us with 181,190 scores from 25,580 children for the analysis. See Table 2 for a detailed description of the cohorts. Polynomial terms for zBMI, zHeight, and zMass were included as covariates in linear mixed models (LMMs) for statistical inference. LMM fits and comparisons were carried out using the Julia programming language [60] with the *MixedModels.jl* package [61, 62]; partial-fitted effects were calculated using the *MixedModelsExtras.jl* package [63].

**Table 2:** Detailed description of sample

Measurement	Number of children	Number of events	Number of observations
Pre-pandemic 2012–2020	16,395	825	117,897
Pandemic (2021–2023)	9,185	540	63,293
Total	25,580	1,344	181,190

### Regression Discontinuity Design (RDD)

Data was collected from nine pre-pandemic cohorts from 2012 to 2020, and from two pandemic cohorts in 2021 and 2023. We used the COVID pandemic as a quasi-experiment and integrated it into the LMM. The RDD provides statistics that reference a critical date and thus are able to indicate a possible pandemic effect [64, 65]. We set the critical date at 2020–12–31 and dummy coded a pre-pandemic and pandemic cohort variable. Test dates were centred around the critical date. For the pre-pandemic years, i.e. 2012 to 2020, test dates were from the middle of the year; for the COVID pandemic years (2021 to 2023), the specific month of testing was also recorded, which we included to increase the precision of estimates for the 2021–2023 trends. The RDD allowed us to estimate linear pre-pandemic and pandemic slopes and their intercepts at the critical date. The difference between the intercepts estimates the size of the pandemic’s effect at the critical date. The difference between the pre-pandemic and pandemic regression slopes is tested with the interaction of the test date with a 0/1-dummy variable coding pre-pandemic vs. pandemic dates. Thus, significant interactions indicate that trends across years differed before and after the critical date, in other words, that the COVID pandemic affected the secular trends. This is a quasi-experimental approach. Obviously, we do not know what the secular trends would have been without the COVID pandemic.

### Linear Mixed Model Selection

Starting with a version of the LMM reported by Bähr et al. [66], we followed recommendations of Bates et al. [67] and Matuschek et al. [68] for selecting a random effect structure (RES) that was not overparameterised, meaning that (a) the variance–covariance matrix of random-effect terms was not singular and (b) a principal component analysis of the matrix showed unique variance for all variance components (VCs). The further selection of terms was based on the Akaike information criterion (AIC), when the terms under consideration were theoretically motivated; otherwise they were selected based on the Bayesian information criterion (BIC). This complex LMM was overparameterised because of high correlations between the three COVID-related CPs. Dropping the PLZ-related CPs post-pandemic slope led to a parsimonious LMM supported by the data. The RES of the final parsimonious LMM estimated 93 parameters.

In the fixed-effect part, we started with main-effect and bivariate interaction terms of cohort/COVID, sex, linear and quadratic trends of age, and body constitution (linear, quadratic, and cubic trends of bmi; linear and quadratic trends of height and mass) for all eight items. Interaction terms were retained for all items if they were significant for one of them. Conversely, interaction terms that were not significant for any of the items were removed. This was the case for the quadratic trends of height and mass. Removing other quadratic model terms significantly decreased the goodness of fit according to the AIC, yielding a total of 18 × 8 = 144 fixed-effect parameters. Thus, the total number of model parameters (i.e., number of model degrees of freedom) for the parsimonious LMM was 93 + 144 = 237. Its number of effective residual degrees of freedom was 89,282 (i.e., computed as the difference between the number of observations [181,190] and the number of geometric degrees of freedom [91,908] that account for the loss of information due to correlations between Child and PLZ random effects). The effective number of degrees of freedom is considerably smaller than what might be expected given the 180,953 residual degrees of freedom (i.e., difference of 181,190 observations and 237 model parameters), but model fit is still based on around 765 observations per model parameter.

In a re-parameterized post-hoc LMM (i.e., identical goodness of fit), we estimated the significance of the change in slope from pre-pandemic to pandemic cohorts instead of the slope for pandemic cohorts. We will report test statistics from this test in Results when needed. The significance of fixed-effect terms was ascertained by LRTs comparing the reference LMM with drop-one LMMs. Finally, the non-significance of fixed-effect terms not included in the reference LMM was tested by LRTs comparing the reference LMM with add-one LMMs (for details see analysis script in OSF repo).

## Results

### Descriptives

The analysis included 25,580 children (age: 7.56 ± 1.21; height: 130.0 ± 9.0 cm; mass: 28.3 ± 7.0 kg) and 181,190 test scores. Of these children, 49% were girls (n = 12,575) from 251 regions (i.e., zip codes). Appendix Table 4 contains descriptive statistics of test scores in their original metric by sex over the entire measurement period.

#### LMM Fixed Effects

**Fig. 1:**
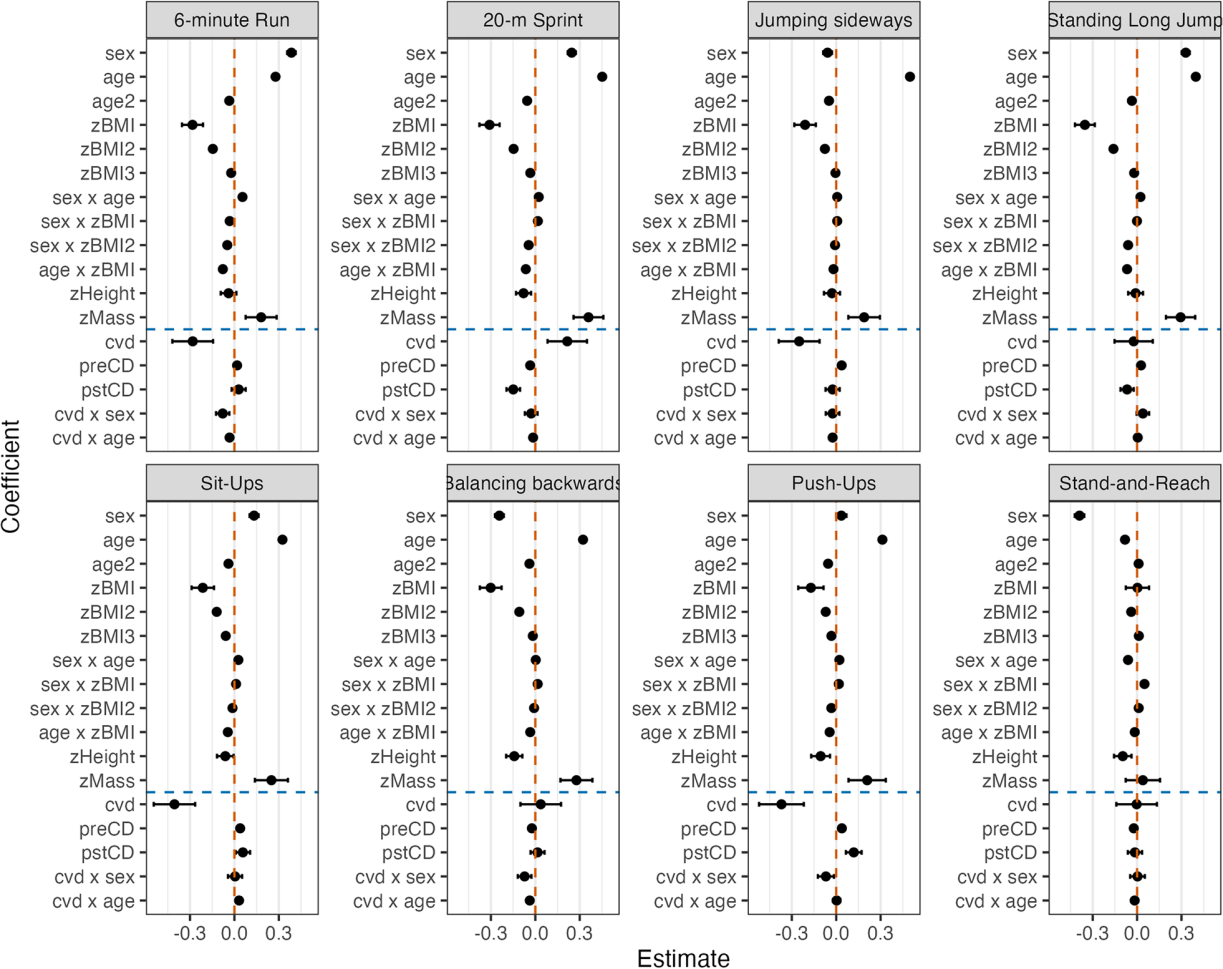
Forest plot of LMM estimates of fixed effects and associated 95% confidence intervals for the eight items

Fixed-effect estimates and associated 95% confidence intervals for the eight items are displayed as a forest plot in Fig. 1; the corresponding test statistics are presented in the Appendix Table 5.

#### Secular Trends and COVID-Pandemic Effects

The RDD provides an analysis of the COVID-pandemic related effects in the context of secular cohort-related trends. Figure 1 shows all fixed effects coefficients and their 95% confidence intervals in z-scores, allowing for comparison.

**Fig. 2:**
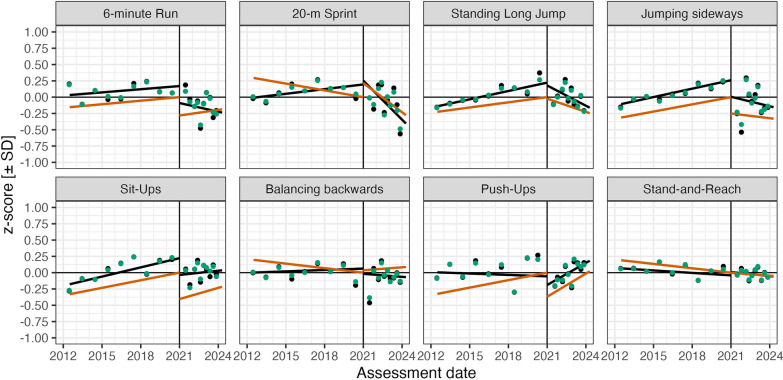
Partial-fitted RDD effects at the assumed critical date of COVID onset (i.e., 2020–12–31; vertical line) for eight items. Black lines: zero-order linear pre-pandemic and pandemic slopes; red lines: corresponding LMM partial-fitted RDD effects; black dots = observed means; most are occluded by green dots = corresponding means of complete LMM predictions (i.e., fitted values). 20 m-Sprint score is speed (i.e., 20 [m/s])

Figure 2 displays the children’s pre-pandemic and pandemic linear trends for the eight PF items for the 2012–2023 cohorts after adjustment of LMM covariates related to age, sex, body constitution, as well as related RES effects. For the 2012–2019 cohorts, mean test dates were the middle of the year; for the 2020–2023 cohorts, information on the test month was available. Therefore, we plot by month within these years to provide a better visualisation of secular trends for the short period of the COVID-pandemic cohorts.

The vertical line marks the RDD critical date (i.e., 2020–12–31) at which the COVID-pandemic change is estimated as the difference between intercepts of linear pre-pandemic and linear pandemic regression lines in the LMM after statistical adjustment for other fixed-effect covariates and RES effects. Black points mark the observed mean z-score for each cohort, mostly occluded by green points, that is, predictions estimated from the complete LMM (i.e., fitted values). Thus, the overall LMM fits the observed means quite well. Red lines visualize partial effects of RDD parameters (i.e., COVID effect at critical date, pre-pandemic and pandemic slopes).

For the *6 min-Run*, a small but significant positive pre-pandemic trend was found (b = 0.02, z = 2.11) and there was a significant negative effect of COVID at the critical date (b = −0.28, z = −4.01). The significant interaction with sex and age show, that this discontinuity was larger for boys (b = −0.08, z = −3.45) and for older children (b = −0.03, z = −3.11). However, there was no evidence for a cohort trend during the pandemic.

The *20 m-Sprint* exhibited a small but significant negative pre-pandemic linear trend (b = −0.04, z = −4.21) that turned into a stronger negative trend for the pandemic cohorts (b = −0.15, z = −6.37). In this case, the intercept at the critical date was estimated significantly positive (b = 0.22, z = 3.18). We interpret this as likely due to an artefactual extrapolation of the strong negative pandemic slope back to the critical date. There was no evidence for any age- or sex-specific interactions with COVID-effects.

*Standing Long Jump* performance increased slightly across the pre-pandemic cohorts (b = 0.03, z = 3.27) and this trend changed in a significantly negative direction for the pandemic cohorts (b = −0.07, z = −2.99). The intercept of the two regression lines extrapolated at the critical date did not differ significantly. Thus, there was no evidence for a discontinuity in performance at the critical date, however the COVID pandemic was associated with a negative change in direction of the trend. Interactions of age and sex were not significant.

For *Jumping sideways* there was a significant, positive, linear, pre-pandemic cohort trend (b = 0.04, z = 4.29), but no evidence for a trend in the pandemic cohorts. However, performance during the COVID-pandemic was significantly lower when estimated at the critical date (b = −0.25, z = −3.56). This was even more pronounced for older children (b = −0.02, z = −2.22), but did not differ for sex. Thus, statistically, there was a significant discontinuity with a downward shift of the positive trend at the critical date.

*Sit-Ups* were characterised by a positive linear trend in the pre-pandemic cohorts (b = 0.04, z = 4.60), equally proceeded in the pandemic cohorts (b = 0.06, z = 2.31). However, that started at a much lower level of performance with a significant negative COVID-effect at the critical date (b = −0.41, z = −5.68), with a larger downward shift for younger children (b = 0.03, z = 2.82), but no evidence for an interaction with sex.

For *Balancing backwards* a small but significant negative pre-pandemic trend was found (b = −0.02, z = −2.61), but there was no evidence of pandemic effects or a trend in the pandemic cohorts. However, significant interactions of sex- and age with COVID-effect were found, showing a tendency to a downward shift of performances at critical date for boys (b = −0.07, z = −3.08) and older children (b = −0.04, z = −3.51).

The profile for *Push ups* showed a positive pre-pandemic slope (b = 0.04, z = 4.06), continued to increase significantly during the pandemic years (b = 0.12, z = 4.38). These positive trends were separated by a large drop in performance at the critical date (b = −0.37, z = −4.80). The COVID-effect was stronger for boys (b = −0.07, z = −2.50).

For *Stand-and-Reach*, there was a-significant negative pre-pandemic slope (b = −0.02, z = −2.53), but no evidence for COVID-effect in performances at critical date and pandemic cohorts. In addition, no significant interactions of sex and age were found.

### Age, Sex, and Body Constitution Effects

**Fig. 3:**
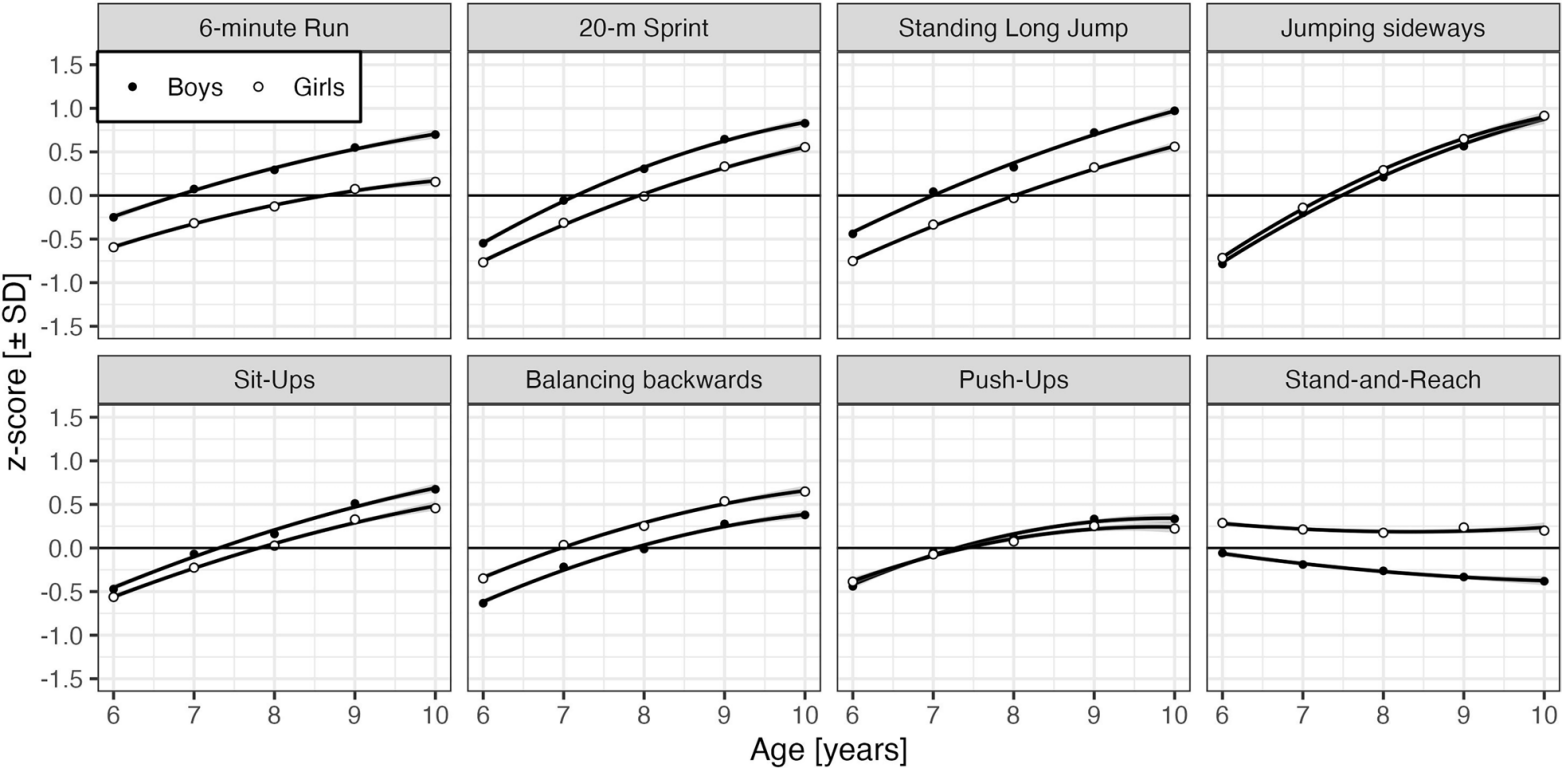
Physical fitness by age and sex for eight items. Continuous lines are zero-order quadratic fits to children’s z-scores; 95%-confidence bands are too narrow for visibility. Each dot is the mean z-score over the means of age bins, representing between 598 and 3,773 children (median: 2,191). The 20 m-Sprint score is speed (i.e., 20 [m/s])

The effects of the COVID pandemic shown in Fig. 2 required adjustment for covariate effects associated with age, sex, and body constitution estimated in the same LMM and shown in the forest plot (Fig. 1). Figure 3 displays observed (i.e., zero-order) age-related developments of performance on items for boys and girls, with higher z-scores always indicating better performance; partial physical fitness scores showed the same profiles. Corresponding estimates of age and sex effects and their interactions, z-statistics, and p-values, adjusted for each other and pandemic-related effects as well as RES effects, are provided in in the Appendix Table 5.

*Effects of age, sex, and age x sex.* As expected, the age effects demonstrate a significant positive linear development across the age range for all items (b = 0.31, z = 28.59 to b = 0.50, z = 53.48), with the exception of Stand-and-Reach, where performance decreases linearly across age range (b = −0.08, z = −8.19). The same was evident as there was a significant slowing of the increase in performance with cross-sectional age for all items (b = −0.06, z = −14.79 to b = −0.04, z = −8.91), except for Stand-and-Reach (b = 0.01, z = 2.37).

**Fig. 4:**
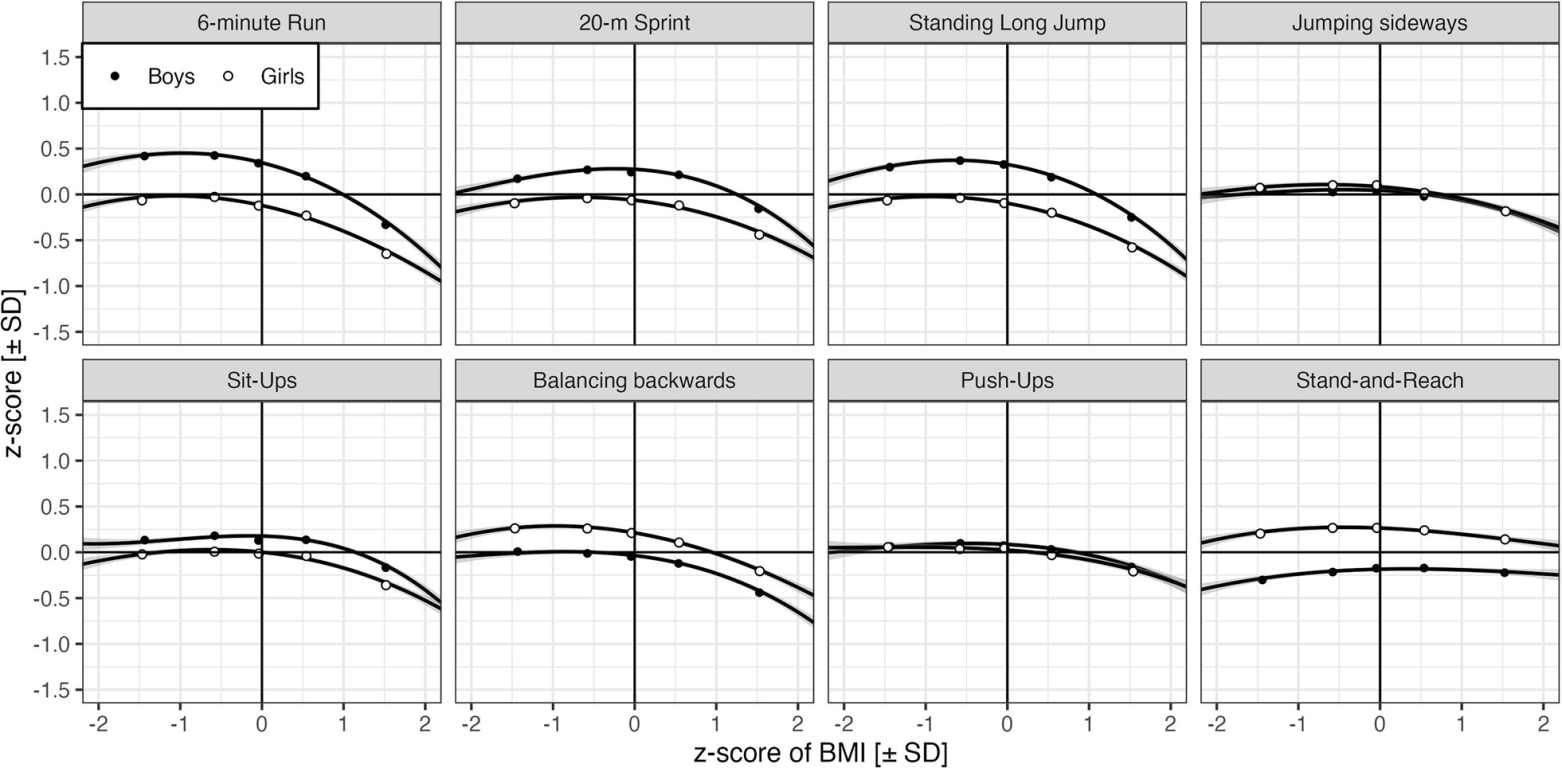
Physical fitness by BMI z-score and sex for the eight items. Continuous lines are zero-order quadratic fits to children’s z-scores and 95%-confidence bands. Each dot is the mean z-score over the means of the BMI z-scores representing between 1,715 and 2,612 children (median: 2,313). The 20m-Sprint score is based on speed (i.e., 20 [m/s])

Generally, sex differences were in expected directions: boys significantly outperformed girls in the 6-min Run, 20 m-Sprint, Standing Long Jump, Sit-Ups, and Push-Ups (b = 0.04, z = 2.00 to b = 0.39, z = 26.43) while girls significantly outperformed boys in Jumping sideways, Balancing backwards and Stand-and-Reach (b = −0.39, z = −23.78 to b = −0.06, z = −3,88). With the exception of Jumping sideways, Balancing backwards, and Push-Ups, these sex-related differences significantly increased with age (|b|= −0.02, |z|= 2.64 to |b|= 0.06, |z|= 6.04. *Effects of body constitution.* Age- and sex-adjusted zBMI effects on physical fitness are shown in Fig. 4. The primary purpose of BMI, mass, and height covariates was “to unveil” cohort-/COVID-specific trends. As expected, a high BMI is associated with strong declines in the 6 min Run, 20 m-Sprint, and Standing Long Jump with much smaller declines for low BMI. The declines in low and high BMI are less pronounced and more symmetric for Jumping sideways, Sit-Ups, Balancing backwards, Push-Ups, and Stand-and-Reach.

The profiles in Fig. 4 are the result not only of quadratic zBMI effects (and their interaction with sex) but also of correlated age- and sex-adjusted quadratic mass and linear height effects. Estimates of these effects and their associated standard errors, z-statistics and p-values are reported in the Appendix Table 5. zBMI was the primary source of the inverted u-shape profiles, in other words, negative quadratic trends were significant for all items (Stand-and-Reach: b = −0.04, z = −6.36 to Standing Long Jump: b = −0.16, z = −29.90). Boys’ and girls’ profiles differed significantly for 6 min Run, 20 m Sprint, Standing Long Jump and Push-Ups (Standing Long Jump: b = −0.06, z = −6.21 to Push-Ups: b = −0.03, z = −2.62) with a stronger impact of BMI for boys. In contrast, there was no evidence for an interaction with sex for Jumping sideways, Sit-Ups, Balancing backwards, and Stand-and-Reach (Jumping Sideways: b = −0.01, z = −0.74 to Sit-Ups: b = −0.01, z = −1.17).

### LMM Correlation Parameters and Zero-Order Correlations for Random Factors

Table 3 contains RES estimates of variance components (VCs) and correlation parameters (CPs) as well as zero-order correlations for the eight items for the two random factors Child and PLZ. For PLZ, we also estimated CPs and zero-order correlations for the COVID effect at the critical date and the pre-pandemic slope, (see Table 3; CPs below the diagonals, zero-order correlations above the diagonals). An estimation of these model parameters primarily guarded against anti-conservative estimates of the fixed effects reported above.

**Table 3:** LMM variance components and correlation parameters (below the diagonals) and zero-order correlations (above the diagonals) for the random factors Child and PLZ

	*SD*	6 min	JSW	20 m	SLJ	SU	BBW	PU	SAR
*Child*									
6 min	0.62		+ 0.38	+ 0.47	+ 0.45	+ 0.34	+ 0.29	+ 0.26	+ 0.05
JSW	0.62	+ 0.49		+ 0.44	+ 0.50	+ 0.40	+ 0.43	+ 0.39	+ 0.08
20 m	0.59	+ 0.61	+ 0.56		+ 0.57	+ 0.40	+ 0.32	+ 0.31	+ 0.07
SLJ	0.61	+ 0.54	+ 0.62	+ 0.80		+ 0.47	+ 0.36	+ 0.33	+ 0.13
SU	0.70	+ 0.39	+ 0.47	+ 0.52	+ 0.56		+ 0.28	+ 0.39	+ 0.10
BBW	0.67	+ 0.34	+ 0.51	+ 0.36	+ 0.44	+ 0.28		+ 0.27	+ 0.16
PU	0.72	+ 0.35	+ 0.55	+ 0.40	+ 0.43	+ 0.52	+ 0.30		+ 0.12
SAR	0.78	+ 0.19	+ 0.22	+ 0.24	+ 0.38	+ 0.20	+ 0.23	+ 0.22	
*PLZ*									
6 min	0.67		+ 0.45	+ 0.45	+ 0.49	+ 0.41	+ 0.37	+ 0.23	+ 0.09
JSW	0.50	+ 0.63		+ 0.51	+ 0.68	+ 0.54	+ 0.62	+ 0.51	+ 0.05
20 m	0.59	+ 0.47	+ 0.46		+ 0.56	+ 0.50	+ 0.41	+ 0.38	+ 0.17
SLJ	0.54	+ 0.57	+ 0.72	+ 0.51		+ 0.69	+ 0.62	+ 0.36	+ 0.19
SU	0.58	+ 0.54	+ 0.70	+ 0.47	+ 0.78		+ 0.42	+ 0.48	+ 0.21
BBW	0.57	+ 0.48	+ 0.58	+ 0.47	+ 0.61	+ 0.57		+ 0.29	+ 0.18
PU	0.53	+ 0.46	+ 0.62	+ 0.46	+ 0.54	+ 0.64	+ 0.43		
SAR	0.45	+ 0.45	+ 0.56	+ 0.59	+ 0.67	+ 0.70	+ 0.61	+ 0.45	+ 0.05
CVD	0.51	−0.54	−0.70	−0.65	−0.84	−0.64	−0.64	−0.47	−0.72
PS	0.07	+ 0.64	+ 0.79	+ 0.68	+ 0.86	+ 0.84	+ 0.61	+ 0.63	+ 0.65

Zero-order correlations above the diagonals are based on pairwise-complete test data. SD: square root of variance component (VC); 6 min: 6 min Run; JSW: Jumping sideways; 20 m: 20 m sprint; SLJ: Standing Long Jump; SU: Sit-Ups; BBW: Balancing backwards; PU: Push-Ups; SaR: Stand and Reach; CVD: covid-effect at critical date; PS: pre-pandemic linear slope; VC for post-pandemic linear slope: 0.08; Residual variance: 0.53

For Child CPs there is a well-defined latent construct of physical fitness comprising a 6-min Run, Jumping sideways, 20 m Sprint, Standing Long Jump, and Sit-Ups with correlation ranging (with one exception) between 0.47 and 0.80. Moreover, Jumping sideways and Standing Long Jump also correlated in this range with Balancing backwards and Push-Ups; Stand-and-Reach did not correlate with the other items. Corresponding zero-order correlations exhibited a similar pattern with smaller correlations ranging from 0.33 to 0.67.

For county (PLZ) all item CPs correlated between 0.43 and 0.78. They correlated negatively with COVID effects (i.e., change at critical date correlated negatively) and positively with pre-pandemic slope. Thus, districts with on average fitter children (indicated by higher scores and larger pre-pandemic slope, i.e., positive or less negative) experienced a larger COVID decrease in performance at the critical date.

## Discussion

Monitoring and surveillance of PF, especially in children, is crucial given its importance and its relationship to numerous health benefits. It has become even more essential as the COVID pandemic influenced life and conditions for physical activities [27]. Even though there have already been several studies on PF trends in recent years, there remains a lack of standardised and long-term monitoring with large datasets in PF research for further specific age groups and samples [16]. This enables to obtain a comprehensive and global evidence of secular trends in the PF of children. Therefore, we conducted an analysis of secular trends in PF between 2012 and 2023 in the federal state of Baden-Wuerttemberg, Germany. Taking advantage of data pooling, we were also able to implement the COVID-19 pandemic as a quasi-experiment and investigate its potential effects.

### Pre-Pandemic Trends in Physical Fitness

For the pre-pandemic cohorts, we found significant, but relatively small trends in both directions for all items during the years under investigation. These trends in PF performance are in line with the latest research findings, which found a weakening of the downward trend occurring at the end of the twentieth century [20, 21].

For endurance, assessed by the 6-min Run, we found a small linear increase before the COVID pandemic starting in 2012. Knowing, that the trend is particularly slight, this finding is fundamentally the same as that of Tomkinson et al. [18], who reported a slowing down of the substantial decline with negligible changes in cardiorespiratory fitness since 2000. Radulović et al. [35] also found a plateauing of performance in the last decade in Slovenian children. The items assessing strength in different parts of the body, were characterised by a slight, positive linear trend for the pre-pandemic cohorts. Equally, Fühner et al. [21] reported a recent upward trend in relative muscle strength up to 2015, however the magnitude of this trend was also small. In Germany, the representative MoMo study reported a consistent level of performance for Standing Long Jump and Push-Ups in all examined age groups, including children aged 4 to 10 years. This supports our findings at a national level [19].

A slight, positive pre-pandemic trend was identified for Jumping sideways, that is the item that tests coordination under time-pressure and combines the abilities of coordination and speed. Interestingly, there was a slight negative pre-pandemic trend in Balancing Backwards and Stand-and-Reach. Balancing Backwards and Stand-and-Reach involve minimal, if any, energy-driven effort, as they primarily relate to information-based abilities like coordination and flexibility, which function as passive systems of energy transfer [69].

It appears that recent efforts to promote physical activity and physical fitness may have started to reverse the previous downward trend, resulting in a slight, positive improvement. However, the findings suggest that these interventions may have primarily focused on enhancing cardiorespiratory and muscular fitness, possibly overlooking the importance of developing more skill-related aspects of physical fitness. While this could explain the observed trends, we cannot definitively say that this is the sole reason, as other factors may also play a role and causal analyses are lacking. The impact of these interventions on coordination remains uncertain and requires further investigation. To promote physical fitness as a multidimensional construct, interventions should develop the wider base of physical fitness and physical activity in a comprehensive and holistic way [70].

Improvement in the tested items as age increases is expected and consistent with recent literature findings [58]. As previously revealed in theoretical concepts, PF performance increased most for children aged 3 to 10 [71]. This age span is extremely important for motor development. Here, goal-oriented and situation-specific movements are developed which form the basis for acquiring a wide range of motor activity and skills. Compared to earlier stages of motor development, movements are characterised by more economy and variability, so that children know better how to use their skills [72]. This underscores the importance of promoting physical fitness early in life through a comprehensive approach that addresses all dimensions. It not only supports general motor development, but also lays the foundation for a wide range of motor skills that either encourage or discourage an individual to be physically active and maintain future PF [7, 73].

### COVID Pandemic Trends in Physical Fitness

The finding that the long-term downward trend in physical fitness has recently stabilized shows that the COVID-19 pandemic was particularly relevant for children’s physical activity routines and levels of PF. By setting a critical date for possible COVID effects and pandemic trends, the results demonstrated a negative influence of the pandemic years. Four items (6-min Run, Jumping Sideways, Sit-Ups, Push-Ups) exhibited a significant negative COVID effect at the critical date. For two items (20-m Sprint, Standing Long Jump), there was a significant negative change from pre-pandemic to pandemic linear trends. In addition, there were three significant interactions of the COVID-effect at the critical date with sex, indicating more negative effect for boys than girls for 6-min Run, Balancing Backwards, and Push-Ups. Finally, interactions with age indicated stronger negative COVID-effects for older children in three items (6-min Run, Jumping Sideways, Balancing Backwards) and a stronger negative COVID-effect for younger children for Sit-Ups.

Our findings suggest that the COVID pandemic had a negative influence on PF levels or interrupted the consistency for six items. Equally, Teich et al. [34] reported lower performance in the 2020–2022 cohorts compared to the pre-pandemic cohorts and estimated that these children would experience developmental delays of approximately 5 months with respect to endurance.

The negative trend for pandemic cohorts was evident for endurance and speed. From a sports science perspective, endurance requires long-term, systematic, and high-intensity training to achieve and sustain a certain level of performance [29, 74]. Additionally, much space and competition with peers is supportive to train endurance and the cardiorespiratory system that determines it. Therefore, children were particularly vulnerable to restrictions and limitations affecting physical activity routines, like the closure of sport clubs or schools [75]. The persistent negative trend, even after the restrictions were lifted, also shows that these negative COVID effects in children have not yet been reversed. It is necessary to implement targeted actions to diminish the developmental loss for children. Furthermore, because endurance and cardiorespiratory fitness is a decisive factor for future health within the PF construct [76, 77]. As stated above, this is even more crucial for children within a specific age range, where the foundations for a lifelong active lifestyle are laid.

For speed, in addition to the large negative change for this trend, there was a discontinuous increment at the critical date, which was surprising. Most likely this is a methodological issue related to the setting of the critical date and not an actual objective estimate. Teich et al. [34] set the critical date as the beginning of the school year and all children were tested from September to November. Nevertheless, it is difficult to determine an exact date, because we do not know when the effects of the COVID pandemic on PF precisely occurred. These effects may also differ between the dimensions, and it cannot be ruled out that age and sex are influencing factors, as well.

The slight, positive pre-pandemic trend for items that assess upper limb and trunk strength was interrupted by a negative COVID effect at the critical date, but followed again by a significant positive linear trend for pandemic cohorts. This points to a rebound effect and indicated that strength was less affected. Indeed, the development of PF is a unique long-term process with multidirectional trends and plasticity influenced by additional factors. It therefore seems logical that changes and adaptions do not occur immediately nor to their full extent in the short term [78]. With respect to trends, whether before, during, or after the COVID pandemic, it is crucial to regularly perform follow-up monitoring to obtain comprehensive and clear evidence of the effects. Nevertheless, the direct assessment, even of weak changes and slight tendencies, could already provide evidence for the need for immediate interventions and action and help to diminish the impact.. The findings indicate that the COVID pandemic did have an influence, causing the level of performance to decline briefly. However the children were obviously able to compensate the drop quite quickly. Strength appears to be a more resilient dimension in relation to the negative effects of the COVID-19 pandemic. The limited exercising opportunities due to the cancellation of organised forms of exercise in sports clubs or physical education lessons primarily affected dimensions other than strength. Doing exercises which reinforce in particular strength, can be easily done at home and with online workouts. This was still possible during the pandemic and may buffered the effects. Teich et al. [34] also reported an upturn in performance from 2021 to 2022 after an initial decline.

During the COVID pandemic, the ways of being physically active changed and the amount of non-organised physical activity increased as a result of the restrictions. More precisely, the time spent on habitual physical activity and playing outside was higher than before the COVID pandemic [79]. It is well known that even the activities of daily life are not just minimal demands and strains on children’s performance, but have a level of intensity that requires and promotes PF [80].

For the item Balancing Backwards related to coordination and Stand-and-Reach for flexibility, we found no evidence of changes in pandemic trends and COVID-effects. The assessment of coordination reveals some methodological issues and these are obvious in recent studies on trends. As an important dimension of the health-oriented PF concept, it is important to measure coordination, but this is challenging because of its complex and multidimensional combination of muscular, sensorial and cognitive components. Skill-related tasks appear to have been less affected by negative influences compared to health-related dimensions during the COVID pandemic, as the multifaceted nature of coordination allows for better mitigation through information-oriented aspects, such as cognitive processes [80].

The observed differences for interactions with sex can be attributed to a range of biological, social, and environmental factors. These mechanisms likely continued to operate—and may even have been amplified—during the COVID-19 pandemic, a period marked by significant disruptions to daily routines, physical activity opportunities, and social structures. The overlapping and interacting mechanisms during the pandemic make it difficult to isolate their specific impact within the scope of our study design.

### BMI and Physical Fitness

There are specific sex-related aspects for coordination, as girls outperform boys in coordination and flexibility. In contrast, there were benefits for boys and improvement rates were higher over the age span included in the study for the other dimensions of endurance, speed and strength. Another common pattern is obvious with respect to coordination. As Fig. 4 illustrates, the quadratic effects of zBMI and thus inverted u-shaped plots indicate different impacts of BMI on the specific items. The 6 mine Run, 20 m-Sprint and Standing Long Jump are especially influenced by BMI. Previous evidence from other studies has already determined that there is a relationship between being overweight and obese and PF [81–85]. Cochrane et al. [82] found a significantly higher probability that children with a low PF were overweight, while others identified being overweight as a potential covariate of PF [81]. The obvious quadratic trends of BMI in PF indicate an optimum BMI level with a beneficial influence on the level of PF. Our findings confirm that a higher BMI results in a lower PF, especially for endurance, strength, and speed which requires more health-related cardiorespiratory and muscular fitness [18, 86]. Considering the emergent coherency between the different PF variables of obesity and being overweight, as well as physical activity and health, it is crucial to break through the cycle [87, 88]..

### Methodological Considerations

For our analysis, we benefitted from a sufficiently large dataset and a comprehensive sample in the federal state of Baden-Wuerttemberg. This points to the particular need for a standardised and continuous assessment of PF in children. A standardised measurement profile enables consistent annual measurement. While some studies only compared a few individual measurement points, we have had regular data since 2012 within the same uniform sample. This ensures a high degree of comparability over the years and a certain homogeneity, as called for by recent research [16, 89].

However, the present article is based on a cross-sectional ad-hoc sample, collected with a citizen science approach. While this approach allows to receive the wide and big range of data, it entailed methodological challenges. One point is a selection bias, as individuals and their social environment who is more aware of the importance of PF and physical activity may be overrepresented due to their greater interest and motivation. Additionally, instruction effects may occur, as multipliers are not professional researchers, leading to potential inconsistencies in data collection. To address these quality concerns, we developed a statistical solution involving event-based data control. Although we have attempted to address the potential for confounding with these considerations, further mechanisms and controls are need to be implemented in future analyses to ensure the validity of the data and to adequately address selection and instruction effects. When implementing the RDD and COVID-19 pandemic as a quasi-experiment, we set the critical date at 2020–12–31. Even if the first restrictions and closures occurred earlier in Germany, they did not immediately affect PF, as PF is a relatively stable construct [90, 91]. In the 2020 cohort, fewer children were tested compared to previous cohorts and when they were, it was mainly in the second half of the year. Hence, we had no statistical power for an earlier critical date. Overall, our findings did not change substantially and reveal the same qualitative pattern whether the critical date was set earlier or later. Therefore, we state that the estimated pandemic effects are robust.

Our analysis was based on a large dataset made possible through data pooling. With the repository MO|RE data (www.motor-research-data.de), we pooled data from existing datasets into a large sample which allowed us to analyse the impact of the pandemic in more than just a small, limited group. The collaborative collection and centred generation of data from existing studies in the repository makes it possible to cross-nationally derive global trends. MO|RE data is discipline-specific and addresses the specific demands and requirements of data on physical fitness [92]. Due to the specificity of results, it is practical to report and consider the different dimensions and items separately. Most of the studies only examine particular aspects of physical fitness, but it is even more meaningful if a sample has been analysed with regard to all components of fitness to obtain a more universal profile. For reporting trends in children’s PF between different populations and over time, an international collaboration and consensus on assessment tools is needed to strengthen the impact of evidence on public health actions. The implementation of a standardised test protocol with common scalable values and metrics help to identify patterns and regions, i.e. with regard to COVID pandemic changes. However, the data being reported on and analysed are still short-term effects of the COVID-19 pandemic. Further investigations are needed to determine whether this is just a short-term dip that will recover over the next few years, or a long-term bend.

### Limitations

Our study is not without limitations. It focuses mainly on short-term analyses on cross-sectional data, which raises the concern of how the observed effects play out in the long term.

Methodological limitations affect the generalizability of results. Although the analysed data is large and consistent, it does not fulfil the requirement of national representativeness as only an ad hoc sample from the federal state of Baden-Wuerttemberg is included. The specific regional environment and economical circumstances could be a possible explanation, but for our sample causal interpretation is not possible. Moreover, there is a lack of additional information, such as socioeconomic status (SES), environmental factors, and the children’s general physical activity, which could provide important contextual information.

Despite these limitations, the study provides valuable insights into the impact of the COVID-19 pandemic on children’s PF and highlights the need for further long-term studies with longitudinal data to better understand the lasting effects. The strengths of this study are the large amount of data which is collected with a standardised consistent assessment of PF in a uniform sample with continuous measuring points.

## Conclusion

Monitoring PF in children at the population level is crucial. Continuous and long-term analyses of trends are essential, particularly in light of the decline in PF caused by the pandemic. It enables early identification of negative trends, allowing targeted interventions to be planned and implemented. Schools and communities must introduce systematic programmes to promote physical activity and counteract the pandemic-related losses in PF. This is especially important as childhood is the phase of development where the foundation is laid for a healthy and active lifestyle. During this “critical motor learning age” even small changes can have a significant impact on the long-term development of PF. The evidence serves as the basis for policymaking and health strategies in Baden-Wuerttemberg, Germany. It enables an evidence-based and data-driven implementation of programmes and the more effective allocation of resources to improve children’s PF and well-being and, in particular, to mitigate the negative effects of the pandemic. Finally, monitoring raises the awareness of the public and society. Parents, teachers, communities, and relevant actors are informed and can contribute actively to the promotion of a healthy lifestyle. In the long-term, this will lead to a more active and healthier society that is prepared for future challenges.

## Supplementary Information

Supplementary material 1.

## Data Availability

The datasets generated and analysed in this study as well as Julia and R scripts are available from the OSF repository: https://osf.io/g37kd/?view_only=8d1a6b36dae147b4892e99a45759d1c4. The datasets generated and analysed in this study are available from the MO|RE data repository, www.motor-research-data.de

## Appendix

See Tables 4 and 5

**Table 4:** Descriptive statistics for test scores by age and sex

	Age	Sex	Test	N	M	SD	min	max
preCOVID	6–10	Girls	6 min	7,408	877	134	500	1305
			JSW	6,522	26	7	3,5	50
			20 m	7,538	4	1	3	6
			SLJ	7,878	119	21	48	190
			SU	7,524	16	6	0	33
			BBW	7,109	32	10	1	48
			PU	5,890	12	4	1	24
			SaR	7,653	3	6	-16´7	22
COVID	6–10	Girls	6 min	3,871	842	138	500	1314
			JSW	3,633	24	8	1,5	51
			20 m	3,950	4	1	3	6
			SLJ	4,492	117	22	48	188
			SU	3,987	16	6	0	35
			BBW	4,233	31	11	0	48
			PU	3,012	12	4	0	25
			SaR	4,453	3	6	−16´7	22
preCOVID		Boys	6 min	7,755	946	157	500	1431
			JSW	6,773	25	8	1	51
			20 m	7,884	5	1	3	6
			SLJ	8,245	127	22	53	203
			SU	7,925	17	6	0	35
			BBW	7,478	29	10	0	48
			PU	6,354	12	4	0	26
			SaR	7,961	0	6	−19	19
COVID		Boys	6 min	3,847	901	159	510	1404
			JSW	3,592	24	8	3	50
			20 m	3,967	4	1	3	6
			SLJ	4,521	127	23	52	202
			SU	3,997	17	7	0	36
			BBW	4,265	28	11	0	48
			PU	3,010	12	4	0	26
			SaR	4,463	0	6	−20	19

Test scores are rounded without decimal places; JSW: Jumping sideways; SLJ: Standing Long Jump; BBW: Balancing backwards; SaR: Stand and Reach; 6 min: 6 min Run; 20 m: 20 m sprint; SU: Sit-Ups; PU: Push-Ups

**Table 5:** LMM estimates of fixed effects (6–10 year old children)

Test	Coefficient	Estimate	SE	z-statistic	p-value
6-min Run	sex	0.3852	0.0146	26.4276	0.0000
	age	0.2779	0.0091	30.5865	0.0000
	age2	−0.0348	0.0039	−8.9108	0.0000
	zBMI	−0.2832	0.0363	−7.8018	0.0000
	zBMI2	−0.1459	0.0056	−25.9234	0.0000
	zBMI3	−0.0220	0.0095	−2.3198	0.0203
	sex x age	0.0547	0.0092	5.9534	0.0000
	sex x zBMI	−0.0319	0.0102	−3.1156	0.0018
	sex x BMI2	−0.0482	0.0103	−4.6937	0.0000
	age x zBMI	−0.0782	0.0046	−17.0877	0.0000
	zHeight	−0.0395	0.0270	−1.4668	0.1424
	zMass	0.1807	0.0535	3.3769	0.0007
	cvd	−0.2818	0.0702	−4.0118	0.0001
	preCD	0.0181	0.0086	2.1087	0.0350
	pstCD	0.0288	0.0242	1.1926	0.2330
	cvd x sex	−0.0791	0.0229	−3.4504	0.0006
	cvd x age	−0.0328	0.0105	−3.1090	0.0019
20 −m Sprint	sex	0.2460	0.0140	17.6338	0.0000
	age	0.4519	0.0087	52.1313	0.0000
	age2	−0.0553	0.0037	−14.7890	0.0000
	zBMI	−0.3094	0.0348	−8.8945	0.0000
	zBMI2	−0.1467	0.0054	−27.1954	0.0000
	zBMI3	−0.0338	0.0091	−3.7306	0.0002
	sex x age	0.0232	0.0088	2.6519	0.0080
	sex x zBMI	0.0173	0.0098	1.7670	0.0772
	sex x BMI2	−0.0448	0.0098	−4.5598	0.0000
	age x zBMI	−0.0640	0.0044	−14.6976	0.0000
	zHeight	−0.0795	0.0259	−3.0742	0.0021
	zMass	0.3592	0.0513	7.0039	0.0000
	cvd	0.2159	0.0680	3.1765	0.0015
	preCD	−0.0347	0.0082	−4.2045	0.0000
	pstCD	−0.1489	0.0234	−6.3687	0.0000
	cvd x sex	−0.0277	0.0219	−1.2610	0.2073
	cvd x age	−0.0149	0.0100	−1.4835	0.1379
Jumping sideways	sex	−0.0584	0.0151	−3.8794	0.0001
	age	0.4985	0.0093	53.4785	0.0000
	age2	−0.0487	0.0041	−11.9660	0.0000
	zBMI	−0.2103	0.0371	−5.6690	0.0000
	zBMI2	−0.0763	0.0057	−13.2986	0.0000
	zBMI3	−0.0049	0.0097	−0.5080	0.6114
	sex x age	0.0068	0.0095	0.7217	0.4705
	sex x zBMI	0.0067	0.0105	0.6312	0.5279
	sex x BMI2	−0.0078	0.0105	−0.7436	0.4571
	age x zBMI	−0.0183	0.0046	−3.9298	0.0001
	zHeight	−0.0285	0.0277	−1.0294	0.3033
	zMass	0.1887	0.0545	3.4604	0.0005
	cvd	−0.2502	0.0703	−3.5610	0.0004
	preCD	0.0369	0.0086	4.2941	0.0000
	pstCD	−0.0243	0.0243	−0.9980	0.3183
	cvd x sex	−0.0245	0.0236	−1.0378	0.2993
	cvd x age	−0.0243	0.0109	−2.2214	0.0263
Standing Long Jump	sex	0.3286	0.0139	23.6812	0.0000
	age	0.3967	0.0085	46.6573	0.0000
	age2	−0.0352	0.0037	−9.5216	0.0000
	zBMI	−0.3527	0.0342	−10.3149	0.0000
	zBMI2	−0.1598	0.0053	−29.8982	0.0000
	zBMI3	−0.0200	0.0090	−2.2259	0.0260
	sex x age	0.0227	0.0086	2.6378	0.0083
	sex x zBMI	−0.0008	0.0098	−0.0780	0.9378
	sex x BMI2	−0.0606	0.0098	−6.2067	0.0000
	age x zBMI	−0.0677	0.0043	−15.8969	0.0000
	zHeight	−0.0102	0.0256	−0.3988	0.6901
	zMass	0.2938	0.0504	5.8316	0.0000
	cvd	−0.0232	0.0659	−0.3523	0.7246
	preCD	0.0264	0.0081	3.2700	0.0011
	pstCD	−0.0675	0.0226	−2.9944	0.0028
	cvd x sex	0.0392	0.0216	1.8136	0.0697
	cvd x age	0.0042	0.0098	0.4318	0.6659
Sit-Ups	sex	0.1328	0.0155	8.5764	0.0000
	age	0.3246	0.0096	33.7635	0.0000
	age2	−0.0402	0.0041	−9.7037	0.0000
	zBMI	−0.2137	0.0386	−5.5387	0.0000
	zBMI2	−0.1198	0.0060	−19.9422	0.0000
	zBMI3	−0.0586	0.0101	−5.8064	0.0000
	sex x age	0.0267	0.0097	2.7405	0.0061
	sex x zBMI	0.0111	0.0109	1.0171	0.3091
	sex x BMI2	−0.0128	0.0110	−1.1653	0.2439
	age x zBMI	−0.0446	0.0049	−9.1694	0.0000
	zHeight	−0.0614	0.0287	−2.1391	0.0324
	zMass	0.2500	0.0569	4.3911	0.0000
	cvd	−0.4051	0.0714	−5.6755	0.0000
	preCD	0.0389	0.0084	4.6030	0.0000
	pstCD	0.0572	0.0248	2.3098	0.0209
	cvd x sex	0.0044	0.0243	0.1825	0.8552
	cvd x age	0.0313	0.0111	2.8161	0.0049
Balancing Backwards	sex	−0.2426	0.0153	−15.8359	0.0000
	age	0.3223	0.0095	34.0890	0.0000
	age2	−0.0397	0.0041	−9.7882	0.0000
	zBMI	−0.3014	0.0374	−8.0592	0.0000
	zBMI2	−0.1082	0.0058	−18.6047	0.0000
	zBMI3	−0.0168	0.0098	−1.7130	0.0867
	sex x age	0.0030	0.0094	0.3190	0.7497
	sex x zBMI	0.0161	0.0107	1.5149	0.1298
	sex x BMI2	−0.0085	0.0106	−0.7968	0.4256
	age x zBMI	−0.0344	0.0047	−7.3869	0.0000
	zHeight	−0.1420	0.0279	−5.0830	0.0000
	zMass	0.2779	0.0551	5.0440	0.0000
	cvd	0.0367	0.0700	0.5240	0.6003
	preCD	−0.0231	0.0088	−2.6106	0.0090
	pstCD	0.0152	0.0241	0.6312	0.5279
	cvd x sex	−0.0722	0.0235	−3.0761	0.0021
	cvd x age	−0.0376	0.0107	−3.5058	0.0005
Push-Ups	sex	0.0347	0.0175	1.9884	0.0468
	age	0.3121	0.0109	28.5899	0.0000
	age2	−0.0547	0.0048	−11.4757	0.0000
	zBMI	−0.1710	0.0436	−3.9202	0.0001
	zBMI2	−0.0708	0.0068	−10.4350	0.0000
	zBMI3	−0.0324	0.0114	−2.8479	0.0044
	sex x age	0.0215	0.0112	1.9152	0.0555
	sex x zBMI	0.0178	0.0124	1.4380	0.1504
	sex x BMI2	−0.0324	0.0124	−2.6240	0.0087
	age x zBMI	−0.0444	0.0055	−8.0129	0.0000
	zHeight	−0.1052	0.0324	−3.2478	0.0012
	zMass	0.2093	0.0642	3.2587	0.0011
	cvd	−0.3699	0.0771	−4.7989	0.0000
	preCD	0.0382	0.0094	4.0621	0.0000
	pstCD	0.1186	0.0271	4.3799	0.0000
	cvd x sex	−0.0695	0.0278	−2.5013	0.0124
	cvd x age	0.0031	0.0128	0.2400	0.8104
Stand-and-Reach	sex	−0.3890	0.0164	−23.7798	0.0000
	age	−0.0816	0.0100	−8.1913	0.0000
	age2	0.0103	0.0043	2.3648	0.0180
	zBMI	0.0024	0.0401	0.0586	0.9533
	zBMI2	−0.0398	0.0063	−6.3548	0.0000
	zBMI3	0.0117	0.0105	1.1064	0.2686
	sex x age	−0.0612	0.0101	−6.0417	0.0000
	sex x zBMI	0.0499	0.0115	4.3453	0.0000
	sex x BMI2	0.0108	0.0115	0.9458	0.3443
	age x zBMI	−0.0157	0.0050	−3.1460	0.0017
	zHeight	−0.0967	0.0301	−3.2160	0.0013
	zMass	0.0390	0.0591	0.6595	0.5096
	cvd	−0.0030	0.0700	−0.0430	0.9657
	preCD	−0.0224	0.0089	−2.5245	0.0116
	pstCD	−0.0145	0.0245	−0.5904	0.5549
	cvd x sex	0.0028	0.0254	0.1105	0.9120
	cvd x age	−0.0164	0.0115	−1.4354	0.1512

## Abbreviations

AIC: Akaike Information Criterion
BIC: Bayesian Information Criterion
BMI: Body Mass Index
zBMI: Body Mass Index Standard Deviation Score
COVID: COVID-19 (Coronavirus SARS-CoV-2)
CPs: Correlation Parameters
GMT: German Motor Test 6–18
KITT +: Kinderturntest Plus 3–10
LMM: Linear Mixed Model
PF: Physical Fitness
PLZ: Postal Code (i.e. zip code)
RES: Random Effect Structure
RDD: Regression Discontinuity Design
VCs: Variance Components
