# Supplementary material for: Secular Trends of Children’s Physical Fitness and the Impact of the COVID-Pandemic for Years 2012 to 2023
Source: Sports Med Open. 2025 Jun 24;11:80. doi: 10.1186/s40798-025-00881-2 (PMC12187630; doi:10.1186/s40798-025-00881-2)
Supplement: Supplementary file 1 — Supplementary material 1. [file 40798_2025_881_MOESM1_ESM.docx]

# Supplementary Material

# Secular Trends of Children’s Physical Fitness and the Impact of the COVID-Pandemic for Years 2012 to 2023

Tanja Eberhardt^1^, Klaus Bös^1^, Alexander Woll^1^, Reinhold Kliegl^2^, & Claudia Niessner^1^

^1^ Karlsruhe Institute of Technology, Institute of Sports and Sports Science, Karlsruhe, Germany

^2^ University of Potsdam, Division of Training and Movement Sciences, Potsdam, Germany

Corresponding author:

Tanja Eberhardt, [tanja.eberhardt@kit.edu](mailto:tanja.eberhardt@kit.edu), ORCID-ID: <https://orcid.org/0009-0001-0992-060X>

**Supplementary Material**


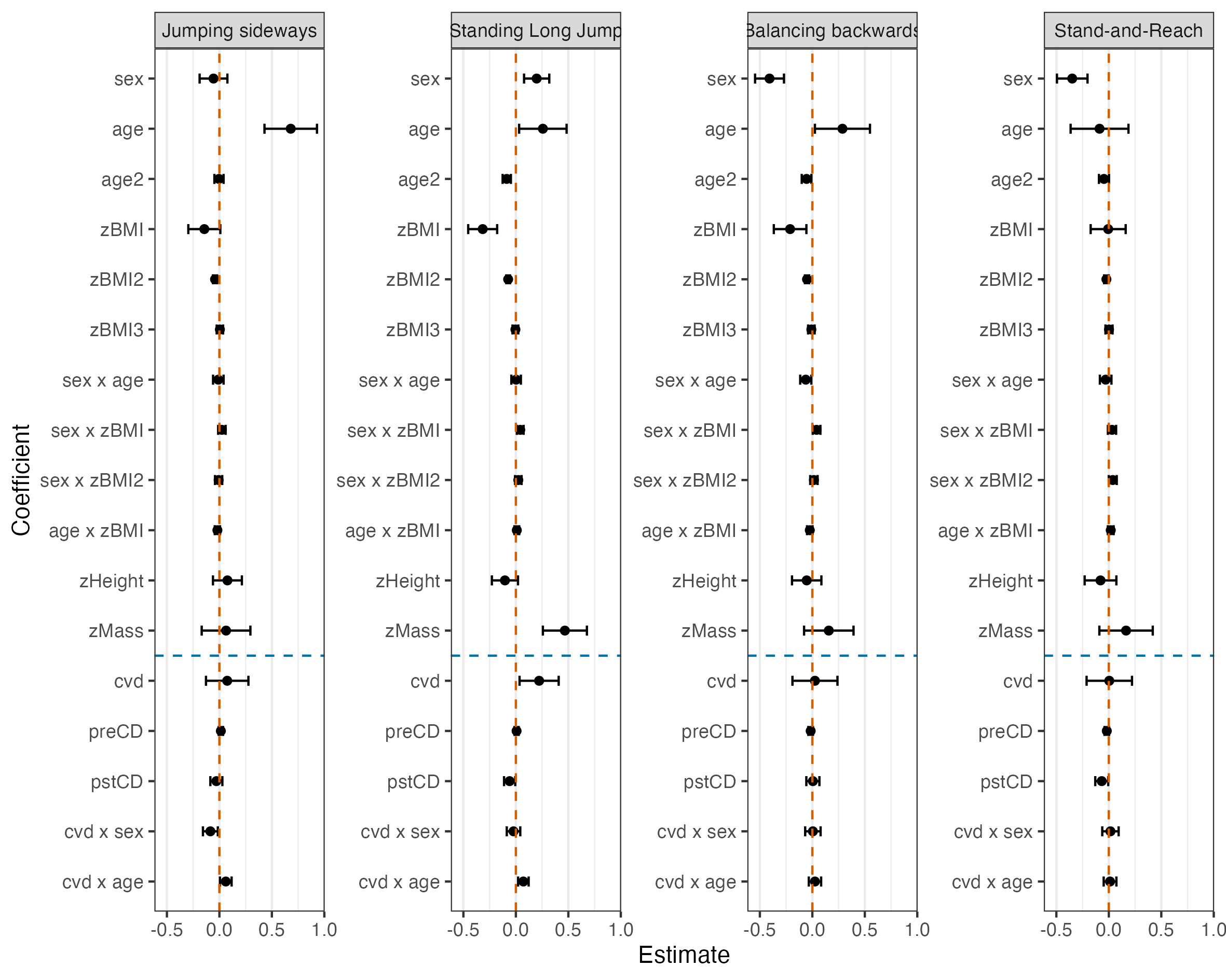


***Supplementary Figure 1.*** *Forest plot of LMM estimates of fixed effects and associated 95% confidence intervals for the four items (3-5 year old children).*


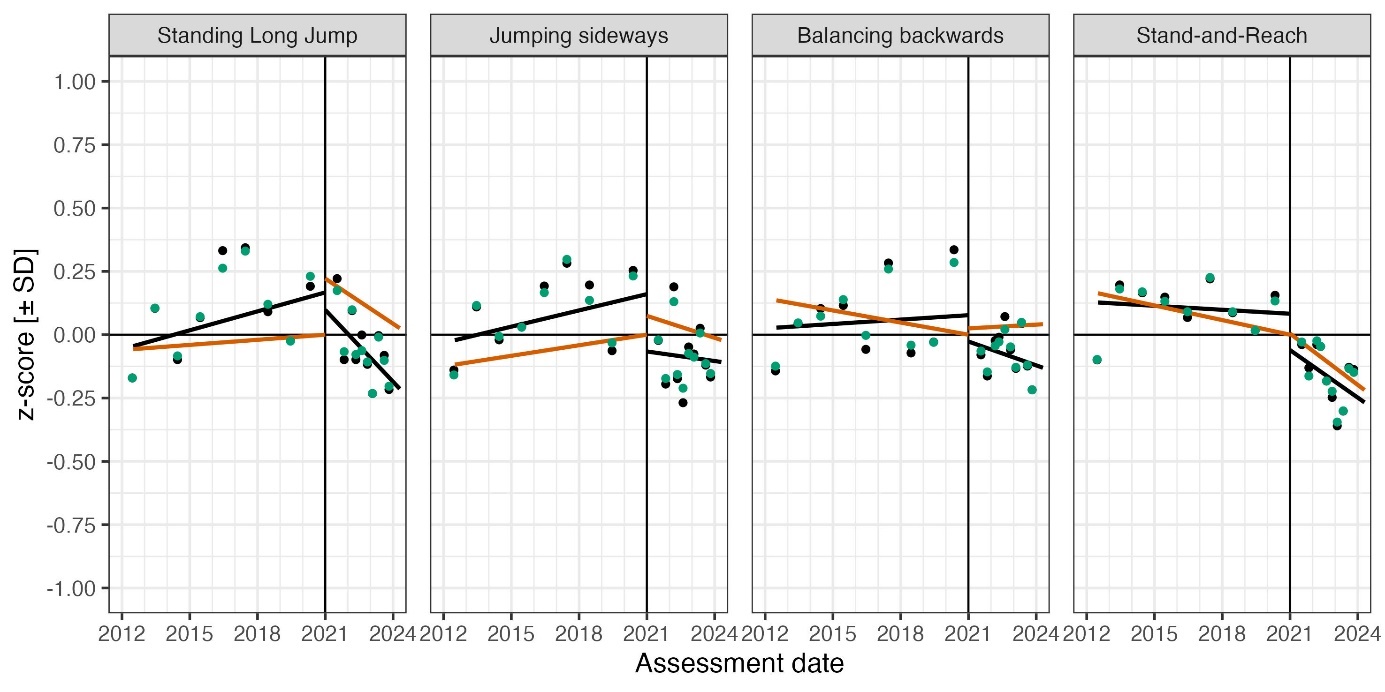


***Supplementary Figure 2.*** *Partial-fitted RDD effects at the assumed critical date of COVID onset (i.e., 2020-12-31; vertical line) for the four items (3-5 year old children). Black lines: zero-order linear pre-pandemic and pandemic slopes; red lines: corresponding LMM partial-fitted RDD effects; black dots = observed means; most are occluded by green dots = corresponding means of complete LMM predictions (i.e., fitted values).*


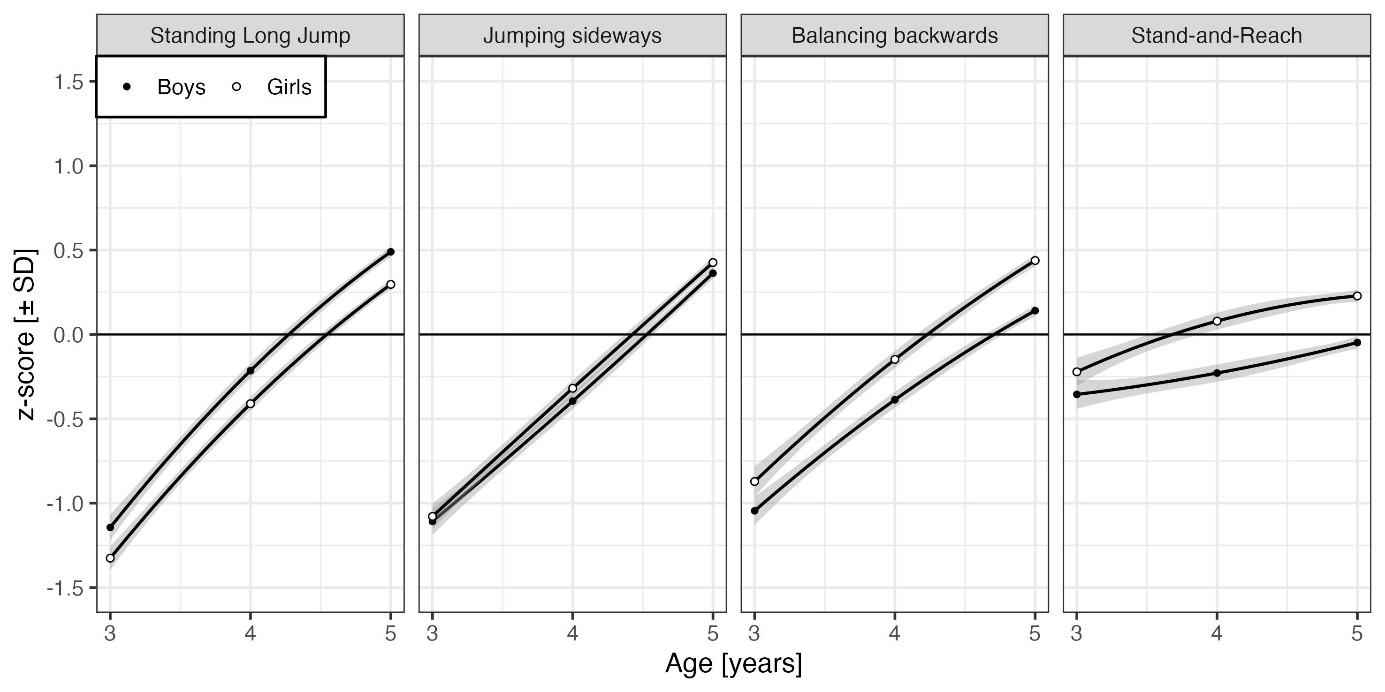


***Supplementary Figure 3.*** *Physical fitness by age and sex for the four items (3-5 year old children). Continuous lines are zero-order quadratic fits to children’s z-scores; 95%-confidence bands are too narrow for visibility.*


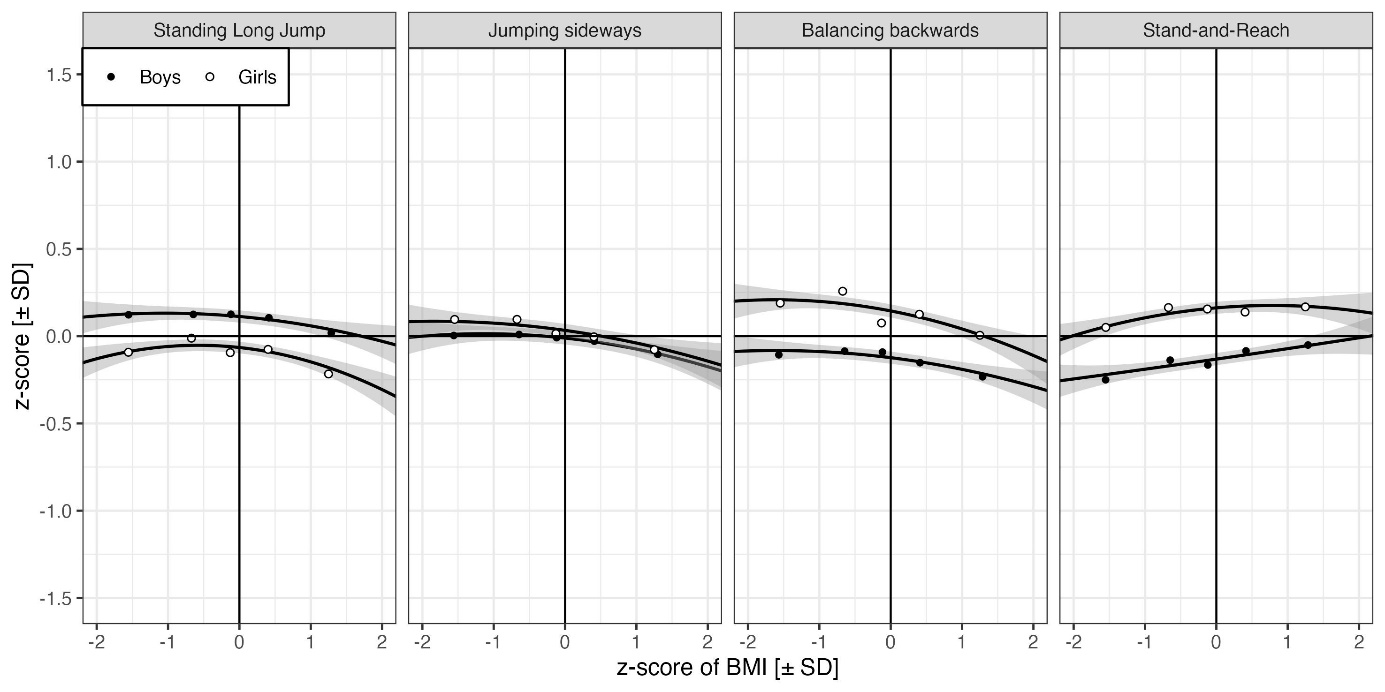


***Supplementary Figure. 4*** *Physical fitness by BMI z-score and sex for the four items (3-5 year old children). Continuous lines are zero-order quadratic fits to children’s z-scores and 95%-confidence bands.*

***Supplementary Table 1.*** *LMM estimates of fixed effects (3-5 year old children)*

| **Test** | **Coefficient** | **Estimate** | | **SE** | **z-statistic** | **p-value** | |
| --- | --- | --- | --- | --- | --- | --- | --- |
| Jumping sideways | sex | -0.0561 | | 0.0675 | -0.8309 | 0.4061 |  |
|  | age | 0.6798 | | 0.1276 | 5.3277 | 0.0000 |  |
|  | age2 | -0.0039 | | 0.0223 | -0.1765 | 0.8599 |  |
|  | zBMI | -0.1436 | | 0.0778 | -1.8445 | 0.0651 |  |
|  | zBMI2 | -0.0414 | | 0.0100 | -4.1496 | 0.0000 |  |
|  | zBMI3 | 0.0048 | | 0.0156 | 0.3069 | 0.7589 |  |
|  | sex x age | -0.0102 | | 0.0256 | -0.4004 | 0.6888 |  |
|  | sex x zBMI | 0.0238 | | 0.0181 | 1.3186 | 0.1873 |  |
|  | sex x BMI2 | -0.0074 | | 0.0176 | -0.4226 | 0.6726 |  |
|  | age x zBMI | -0.0190 | | 0.0128 | -1.4819 | 0.1384 |  |
|  | zHeight | 0.0765 | | 0.0703 | 1.0876 | 0.2768 |  |
|  | zMass | 0.0634 | | 0.1184 | 0.5352 | 0.5925 |  |
|  | cvd | 0.0744 | | 0.1031 | 0.7221 | 0.4703 |  |
|  | preCD | 0.0139 | | 0.0108 | 1.2800 | 0.2006 |  |
|  | pstCD | -0.0289 | | 0.0292 | -0.9900 | 0.3222 |  |
|  | cvd x sex | -0.0858 | | 0.0362 | -2.3706 | 0.0178 |  |
|  | cvd x age | 0.0610 | | 0.0283 | 2.1567 | 0.0310 |  |
| Standing Long Jump | sex | | 0.1982 | 0.0613 | 3.2341 | 0.0012 | |
|  | age | | 0.2572 | 0.1154 | 2.2300 | 0.0257 | |
|  | age2 | | -0.0868 | 0.0201 | -4.3092 | 0.0000 | |
|  | zBMI | | -0.3162 | 0.0704 | -4.4893 | 0.0000 | |
|  | zBMI2 | | -0.0736 | 0.0090 | -8.1550 | 0.0000 | |
|  | zBMI3 | | -0.0044 | 0.0141 | -0.3121 | 0.7549 | |
|  | sex x age | | 0.0023 | 0.0232 | 0.1003 | 0.9201 | |
|  | sex x zBMI | | 0.0417 | 0.0164 | 2.5436 | 0.0110 | |
|  | sex x zBMI2 | | 0.0233 | 0.0159 | 1.4629 | 0.1435 | |
|  | age x zBMI | | 0.0090 | 0.0117 | 0.7735 | 0.4393 | |
|  | zHeight | | -0.1042 | 0.0635 | -1.6401 | 0.1010 | |
|  | zMass | | 0.4671 | 0.1070 | 4.3650 | 0.0000 | |
|  | cvd | | 0.2216 | 0.0951 | 2.3304 | 0.0198 | |
|  | preCD | | 0.0067 | 0.0102 | 0.6576 | 0.5108 | |
|  | pstCD | | -0.0594 | 0.0272 | -2.1849 | 0.0289 | |
|  | cvd x sex | | -0.0223 | 0.0328 | -0.6802 | 0.4964 | |
|  | cvd x age | | 0.0714 | 0.0257 | 2.7831 | 0.0054 | |
| Balancing Backwards | sex | -0.4079 | | 0.0703 | -5.8008 | 0.0000 | |
|  | age | 0.2868 | | 0.1338 | 2.1439 | 0.0320 | |
|  | age2 | -0.0551 | | 0.0235 | -2.3395 | 0.0193 | |
|  | zBMI | -0.2119 | | 0.0792 | -2.6743 | 0.0075 | |
|  | zBMI2 | -0.0506 | | 0.0102 | -4.9535 | 0.0000 | |
|  | zBMI3 | -0.0094 | | 0.0159 | -0.5893 | 0.5556 | |
|  | sex x age | -0.0628 | | 0.0270 | -2.3273 | 0.0199 | |
|  | sex x zBMI | 0.0403 | | 0.0186 | 2.1649 | 0.0304 | |
|  | sex x BMI2 | 0.0150 | | 0.0180 | 0.8302 | 0.4064 | |
|  | age x zBMI | -0.0242 | | 0.0135 | -1.7889 | 0.0736 | |
|  | zHeight | -0.0538 | | 0.0714 | -0.7542 | 0.4507 | |
|  | zMass | 0.1568 | | 0.1202 | 1.3044 | 0.1921 | |
|  | cvd | 0.0249 | | 0.1094 | 0.2272 | 0.8203 | |
|  | preCD | -0.0160 | | 0.0113 | -1.4138 | 0.1574 | |
|  | pstCD | 0.0050 | | 0.0318 | 0.1568 | 0.8754 | |
|  | cvd x sex | 0.0053 | | 0.0375 | 0.1426 | 0.8866 | |
|  | cvd x age | 0.0250 | | 0.0300 | 0.8333 | 0.4046 | |
| Stand-and-Reach | sex | -0.3487 | | 0.0743 | -4.6939 | 0.0000 | |
|  | age | -0.0884 | | 0.1406 | -0.6286 | 0.5296 | |
|  | age2 | -0.0461 | | 0.0245 | -1.8795 | 0.0602 | |
|  | zBMI | -0.0062 | | 0.0851 | -0.0726 | 0.9421 | |
|  | zBMI2 | -0.0231 | | 0.0110 | -2.1061 | 0.0352 | |
|  | zBMI3 | 0.0018 | | 0.0172 | 0.1065 | 0.9152 | |
|  | sex x age | -0.0301 | | 0.0281 | -1.0711 | 0.2841 | |
|  | sex x zBMI | 0.0303 | | 0.0200 | 1.5201 | 0.1285 | |
|  | sex x BMI2 | 0.0374 | | 0.0194 | 1.9267 | 0.0540 | |
|  | age x zBMI | 0.0183 | | 0.0141 | 1.2907 | 0.1968 | |
|  | zHeight | -0.0792 | | 0.0771 | -1.0279 | 0.3040 | |
|  | zMass | 0.1643 | | 0.1298 | 1.2651 | 0.2058 | |
|  | cvd | 0.0045 | | 0.1104 | 0.0408 | 0.9675 | |
|  | preCD | -0.0192 | | 0.0112 | -1.7208 | 0.0853 | |
|  | pstCD | -0.0675 | | 0.0315 | -2.1427 | 0.0321 | |
|  | cvd x sex | 0.0155 | | 0.0398 | 0.3902 | 0.6964 | |
|  | cvd x age | 0.0117 | | 0.0309 | 0.3765 | 0.7065 | |

*Supplementary Table 2. LMM variance components and correlation parameters (below the diagonals) and zero-order correlations (above the diagonals) for the random factors Child and PLZ (3-5 year old children)*

|  | *SD* | JSW | SLJ | BBW | SAR |
| --- | --- | --- | --- | --- | --- |
| **Child** |  |  |  |  |  |
| JSW | 0.63 |  | +0.55 | +0.45 | +0.21 |
| SLJ | 0.59 | +0.64 |  | +0.39 | +0.20 |
| BBW | 0.66 | +0.51 | +0.40 |  | +0.18 |
| SAR | 0.77 | +0.22 | +0.29 | +0.18 |  |
| **PLZ** |  |  |  |  |  |
| JSW | 0.40 |  | +0.66 | +0.48 | +0.35 |
| SLJ | 0.41 | +0.68 |  | +0.50 | +0.41 |
| BBW | 0.50 | +0.36 | +0.43 |  | +0.23 |
| SAR | 0.40 | +0.51 | +0.58 | +0.25 |  |
| CVD | 0.26 | -0.69 | -0.71 | -0.06 | -0.58 |
| PS | 0.07 | +0.67 | +0.82 | +0.49 | +0.62 |

Zero -order correlations above the diagonals are based on pairwise-complete test data. SD: square root of variance component (VC); 6min: 6-minute Run; JSW: Jumping sideways; 20m: 20 m sprint; SLJ: Standing Long Jump; SU: Sit-Ups; BBW: Balancing backwards; PU: Push-Ups; SaR: Stand and Reach; CVD: covid-effect at critical date; PS: pre-pandemic linear slope; VC for post-pandemic linear slope: 0.06; Residual variance: 0.51.
